# Efficacy and safety of brain radiotherapy combined with antibody-drug conjugates in patients with HER2-positive breast cancer brain metastases

**DOI:** 10.3389/fonc.2026.1899301

**Published:** 2026-08-03

**Authors:** Dongxing Shen, Deyou Kong, Longyu Zhu, Zhikun Liu, Jun Zhang

**Affiliations:** Department of Radiation Oncology, The Fourth Hospital of Hebei Medical University, Shijiazhuang, Hebei, China

**Keywords:** antibody-drug conjugates, brain metastases, breast cancer, HER2, radiotherapy

## Abstract

Antibody-drug conjugates (ADCs) have shown promising efficacy in patients with human epidermal growth factor receptor 2 (HER2)-positive breast cancer and brain metastases. This study aimed to investigate the efficacy and safety of brain radiotherapy combined with ADCs in patients with HER2-positive breast cancer and brain metastases in routine Chinese clinical practice. We retrospectively analyzed data from 35 patients with HER2-positive breast cancer and brain metastases treated with brain radiotherapy plus trastuzumab deruxtecan (T-DXd) or trastuzumab emtansine (T-DM1) at the Department of Radiation Oncology, The Fourth Hospital of Hebei Medical University, between January 2023 and January 2025. Most patients had prior pyrotinib treatment. Intracranial progression-free survival (iPFS), progression-free survival (PFS), overall survival (OS), and treatment safety were analyzed. Median follow-up was 15.7 months (interquartile range [IQR], 8.7–20.8). Median iPFS was 8.4 months (95% confidence interval [CI] 6.9–9.8), median PFS was 7.9 months (95% CI 5.9–10.4), and median OS was 11.8 months (95% CI 8.6–13.8). Intracranial objective response rate (ORR) was 74.3%, and intracranial disease control rate (DCR) was 94.3%. Median iPFS was 7.6 months in the T-DM1 group versus 10.9 months in the T-DXd group (P < 0.001); median PFS was 6.5 months versus 9.7 months (P = 0.021); median OS was 10.4 months versus 12.9 months (P = 0.046). The most common grade ≥3 adverse events were decreased appetite (23%), fatigue (11%), leukopenia (11%), and thrombocytopenia (9%). Among adverse events of special interest, radiation necrosis occurred in 9% (3/35), all were grade 1–2 and occurred exclusively in patients receiving concurrent radiotherapy plus T-DM1; interstitial lung disease occurred in 3% (1/35), grade 1, in the T-DXd group. In Chinese clinical practice, brain radiotherapy combined with either T-DXd or T-DM1 showed promising preliminary efficacy and tolerable safety signals in heavily pyrotinib-pretreated HER2-positive breast cancer patients with brain metastases in this retrospective single-center cohort. In this unadjusted retrospective cohort, patients receiving radiotherapy combined with T-DXd exhibited longer univariate survival outcomes compared with those treated with T-DM1. However, these univariate observational results are preliminary and require prospective multicenter validation with multivariate adjustment.

## 1 Introduction

After lung cancer, breast cancer has become the malignant tumor with the highest risk of brain metastasis (1). Statistically, 30% of patients with breast cancer will eventually develop brain metastases, and approximately 50% of these cases occur in those with the human epidermal growth factor receptor 2 (HER2)-positive subtype (2). Local radiotherapy or surgery is one of the standard treatments for brain metastases in patients with breast cancer (3). However, more than 50% of patients still develop local recurrence or new brain lesions within 1 year after radiotherapy (4). Tyrosine kinase inhibitors (TKIs) have a small molecular weight, can penetrate the blood-brain barrier, and exert definite anti-brain tumor activity, representing an important systemic treatment for breast cancer brain metastases with better efficacy than large-molecule monoclonal antibodies (5, 6). The efficacy of neratinib, tucatinib, lapatinib, pyrotinib, ADCs in breast cancer brain metastases has been confirmed in multiple prospective studies, and radiotherapy may enhance ADC delivery and antitumor efficacy by modulating vascular permeability and/or tumor antigen expression, thereby facilitating drug penetration and target engagement within intracranial lesions (7–10). For patients with stable or tolerably symptomatic HER2-positive active brain metastases, central nervous system (CNS)-active anti-HER2 therapy under close follow-up is also recommended by guidelines and adopted in clinical practice (11). This study aimed to explore the efficacy and safety of brain radiotherapy combined with ADCs in patients with HER2-positive breast cancer and brain metastases. The primary exploratory comparison was between brain radiotherapy combined with T-DXd versus T-DM1, and the secondary exploratory comparison was between concurrent versus sequential administration of ADC and radiotherapy.

## 2 Methods

### 2.1 Patient characteristics

**Figure 1:**
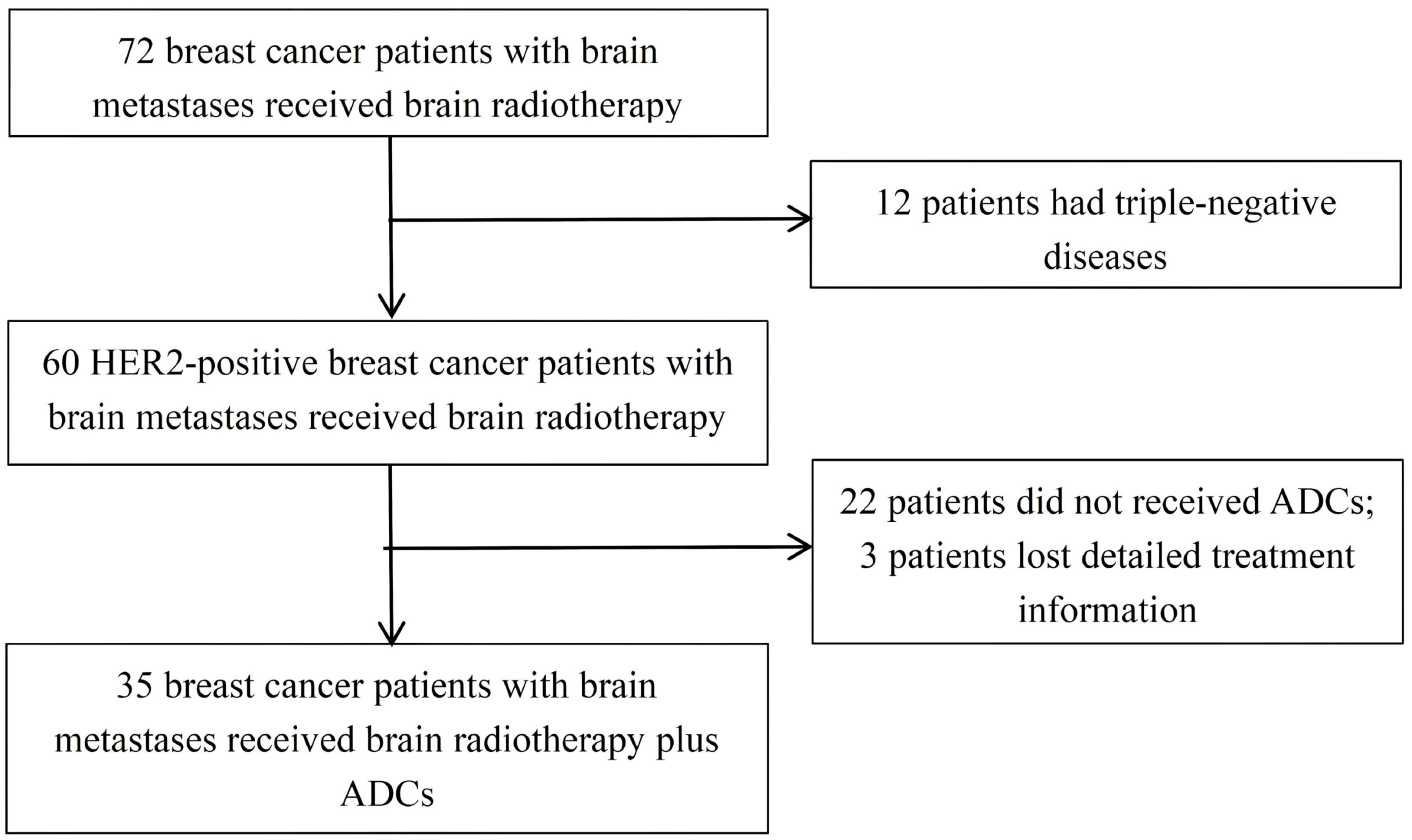
Study flow chart. Keys: HER2: human epidermal growth factor receptor 2; ADCs, antibody-drug conjugates.

We enrolled 72 breast cancer patients with brain metastases who received brain radiotherapy at the Department of Radiation Oncology, The Fourth Hospital of Hebei Medical University, between January 2023 and January 2025. Among them, 60 patients had HER2-positive disease, and 38 received brain radiotherapy plus ADCs. According to inclusion and exclusion criteria, 35 patients were finally included. Inclusion criteria (**Figure 1**): (1) Eastern Cooperative Oncology Group (ECOG) performance status score ≤ 2; (2) life expectancy ≥ 3 months; (3) pathologically confirmed HER2-positive and hormone receptor negative breast cancer with measurable intracranial lesions verified by contrast-enhanced MRI; (4) newly diagnosed brain metastases or intracranial progression of pre-existing brain metastases after prior treatment; (5) brain radiotherapy administered within 90 days before or after ADC initiation, with contrast-enhanced brain MRI repeated every 2–4 months; (6) no contraindications to treatment. Exclusion criteria: (1) pregnant or lactating women; (2) leptomeningeal metastasis; (3) prior treatment with ADCs and brain radiotherapy; (4) incomplete treatment and follow-up data; (5) other concurrent malignancies. This study was approved by the Ethics Committee of The Fourth Hospital of Hebei Medical University (approval number: 2025KS031). Written informed consent was waived per institutional ethics approval due to the retrospective design.

### 2.2 Treatment methods

Before enrollment, 77% had intracranial progression after prior treatment for brain metastases. ADCs included trastuzumab deruxtecan (T-DXd) and trastuzumab emtansine (T-DM1). T-DXd was administered at 5.4 mg/kg intravenously every 21 days; T-DM1 was administered at 3.6 mg/kg intravenously every 21 days. Radiotherapy principles: Patients with ≤ 5 progressive intracranial lesions received stereotactic radiotherapy (SRT) alone; those with > 5 lesions received whole-brain radiotherapy (WBRT) ± SRT. WBRT was delivered at a dose of 30 Gy in 10 fractions using linear accelerator-based three-dimensional conformal radiotherapy or intensity-modulated radiotherapy, and neither hippocampal-avoidance WBRT nor memantine was administered to any patient during the study period. The planning target volume (PTV) was generated by uniformly expanding the whole brain tissue by 3 mm. SRT dose regimens included single-fraction 18–20 Gy, 27 Gy in 3 fractions, 30 Gy in 3 fractions, 27.5 Gy in 5 fractions, or 30 Gy in 5 fractions. SRT fractionation and dose selection followed international guidelines and consensus for brain metastases: single-fraction regimens (18–20 Gy) were exclusively delivered to small lesions with maximum diameter ≤2 cm to maximize local control while limiting normal brain irradiation volume, whereas hypofractionated schedules (27 Gy in 3 fractions, 30 Gy in 3 fractions, 27.5 Gy in 5 fractions, or 30 Gy in 5 fractions) were prioritized for lesions >2 cm or lesions adjacent to critical organs at risk (OARs) to reduce radiation necrosis risk via fractionation-induced normal tissue sparing. No fixed single diameter cutoff was applied; the final schedule integrated lesion size, proximity to optic apparatus/brainstem, total intracranial tumor volume, and extent of peritumoral edema. Uniform institutional dose constraints were enforced across all CyberKnife SRT plans regardless of fraction scheme: for tumor volume, the prescription isodose line covered ≥95% of the PTV; for OARs, single-fraction limits included Dmax <10 Gy for optic nerves/chiasm and Dmax <14 Gy for brainstem, while multi-fraction constraints were adjusted proportionally-Dmax ≤18 Gy for brainstem and ≤17 Gy for visual pathways over 3 fractions, and Dmax ≤25 Gy for optic apparatus and ≤31 Gy for brainstem over 5 fractions. SRT was performed using CyberKnife, with treatment-planning CT images co-registered with contrast-enhanced MRI obtained within 2 weeks before the first treatment. The gross tumor volume (GTV) was defined as metastatic lesions visible on contrast-enhanced CT and MRI. For CyberKnife-based SRT, the PTV was created by uniformly expanding the GTV by 1–2 mm. We referenced published retrospective real-world ADC-radiotherapy cohort studies, which adopted the same time frame to capture all cases of cranial radiotherapy in the peri-ADC period. Concurrent treatment was defined as brain radiotherapy during ADC administration or within 30 days after the last ADC administration; sequential treatment was defined as brain radiotherapy more than 30 days but less than 90 days before or after the last ADC administration. Subsequent local intracranial treatments included WBRT, repeat SRT, and palliative surgical resection; local extracranial treatments included palliative bone radiotherapy and extracranial stereotactic body radiotherapy.

### 2.3 Follow-up and study endpoints

Follow-up was performed via telephone, review of re-hospitalization medical records, and outpatient examination results. Follow-up was closed on February 1, 2026, with a follow-up rate of 100%. The primary endpoint was intracranial progression-free survival (iPFS), defined as the time from ADC initiation to intracranial disease progression, death from any cause, or last follow-up. Secondary endpoints included progression-free survival (PFS), overall survival (OS), intracranial objective response rate (ORR), and safety. PFS was defined as the time from ADC initiation to disease progression or death from any cause. OS was defined as the time from ADC initiation to death from any cause or last follow-up. Short-term efficacy included ORR, complete response (CR), partial response (PR), stable disease (SD), and progressive disease (PD), with ORR = CR rate + PR rate. Safety assessments included clinical symptoms, laboratory tests, and imaging examinations.

### 2.4 Evaluation criteria

Efficacy of intracranial lesions was evaluated using the Response Assessment in Neuro-Oncology Brain Metastases (RANO-BM) criteria. Extracranial lesion efficacy was assessed per Response Evaluation Criteria in Solid Tumors (RECIST) version 1.1. Adverse events were graded according to the Common Terminology Criteria for Adverse Events (CTCAE) version 5.0. Graded prognostic assessment (GPA) for brain metastases was performed using the modified Breast-GPA, including Karnofsky Performance Status score, age, molecular subtype, number of intracranial lesions, and extracranial metastatic status. Radiation necrosis was diagnosed via a standardized multimodal imaging protocol. First, serial follow-up brain imaging consisting of contrast-enhanced MRI, perfusion-weighted imaging, diffusion-weighted imaging, and magnetic resonance spectroscopy was routinely performed to dynamically monitor intracranial lesions. Second, 18F-FDG PET-CT was supplemented for ambiguous lesions to differentiate radiation necrosis from tumor progression, which was implemented in one patient with indeterminate imaging manifestations. Third, all imaging findings were independently reviewed and adjudicated by at least two senior radiologists with specialized neuroimaging expertise. Fourth, therapeutic diagnosis was adopted as auxiliary evidence: lesions with symptomatic relief after intracranial pressure-lowering dehydration and bevacizumab treatment were highly suspected of radiation necrosis.

### 2.5 Statistical analysis

Statistical analysis was performed using SPSS 25.0 software. Continuous variables with non-normal distribution were presented as median and interquartile range (IQR), and categorical variables as frequencies and percentages (%). Between-group comparisons of categorical variables were conducted using the chi-square test. Survival curves were plotted using the Kaplan–Meier method, and differences were compared using the log-rank test. Univariate Cox proportional hazards regression models were constructed to calculate unadjusted hazard ratios (HR) and corresponding 95% confidence intervals (CIs) for all survival endpoints. A two-sided P < 0.05 was considered statistically significant. All statistical tests were performed in accordance with standard biostatistical guidelines to ensure the reliability and validity of the results.

## 3 Results

### 3.1 Patient characteristics

A total of 35 female patients were included, with 107 lesions identified, of which 85 (80%) were < 2 cm in diameter. Median age at brain metastasis diagnosis was 54 years. Five patients (14%) were initially diagnosed with stage IV disease, 3 (9%) had an ECOG score of 2, 27 (77%) had an interval from breast cancer diagnosis to intracranial progression ≥ 36 months, 30 (86%) had received first-line trastuzumab plus pertuzumab and second-line pyrotinib, 29 (83%) had prior anti-HER2 therapy duration ≥ 10 months, 24 (69%) had a Breast-GPA score > 2.5 (stable extracranial disease), 31 (89%) had received ≥ 2 lines of anti-HER2 therapy, 25 (71%) were treated with T-DXd, 23 (66%) had ADC treatment duration ≥ 6 months, 22 (63%) were < 2 cm, 23 (65%) had ≤ 5 brain metastases, 30 (86%) had extracranial metastases, 31 (89%) received SRT ± WBRT, and 22 (63%) received concurrent treatment (**Table 1**).

**Table 1:** Patient characteristics for the whole cohort (n= 35).

Characteristic	n(%)	Characteristic	n (%)
Median age at BM (IQR)	54 (42~60)	ADCs category	
ECOG performance status		T-DM1	10 (29)
0	5 (14)	T-DXd	25 (71)
1	27 (77)	Duration of ADC treatment	
2	3 (9)	≥ 6 months	23 (66)
Initial TNM staging		< 6 months	12 (34)
Stage II	10 (29)	Largest diameter of BM	
Stage III	20 (57)	≥ 2 cm	13 (37)
Stage IV	5 (14)	< 2 cm	22 (63)
Interval between diagnosis of breast cancer and intracranial progression		Number of BM	
< 36 months	8 (23)	1	8 (23)
≥ 36 months	27 (77)	2	4 (11)
Prior anti-HER2 therapy		3–5	11 (31)
Trastuzumab	32 (91)	>5	12 (35)
Pertuzumab	30 (86)	Extracranial metastasis	
Lapatinib	5 (14)	Bone	17 (49)
Pyrotinib	30 (86)	Lung	15 (43)
Duration of prior anti-HER2 therapy		Liver	7 (20)
≥ 10 months	29 (83)	None	5 (14)
< 10 months	6 (17)	Radiotherapy modality	
Lines of anti-HER2 therapy		WBRT alone	4 (11)
≥ 2	31 (89)	WBRT + SRT	8 (23)
< 2	4 (11)	SRT alone	23 (66)
Breast-GPA score		Treatment timing	
≤ 2.5	11 (31)	Concurrent	22 (63)
> 2.5	24 (69)	Sequential	13 (37)

n, number; BM, brain metastases; IQR, interquartile range; HER2, human epidermal growth factor receptor 2; ECOG, Eastern Cooperative Oncology Group; GPA, graded prognostic assessment; ADCs, antibody−drug conjugates; T−DM1, trastuzumab emtansine; T−DXd, trastuzumab deruxtecan; WBRT, whole−brain radiotherapy; SRT, stereotactic radiotherapy.

### 3.2 Survival analysis

**Figure 2:**
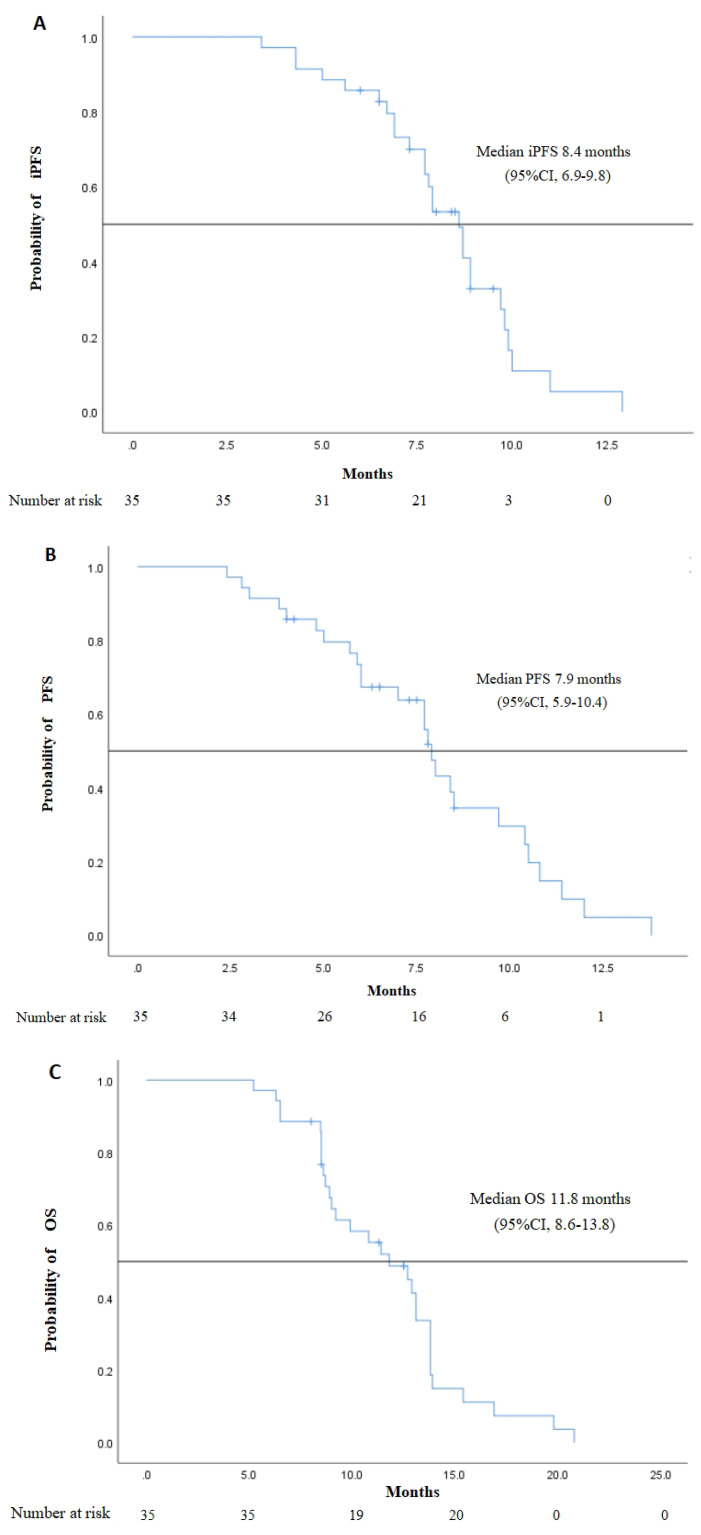
Kaplan–Meier curves for intracranial progression-free survival **(A)**, progression-free survival **(B)**, and overall survival **(C)** in the total cohort. iPFS, intracranial progression-free survival; PFS, progression-free survival; OS, overall survival; CI, confidence interval.

Median follow-up was 15.7 (IQR, 8.7–20.8) months. Median iPFS was 8.4 (95% confidence interval [CI] 6.9–9.8) months, with 6-month and 10-month iPFS rates of 85.7% and 16.4%, respectively. Median PFS was 7.9 (95% CI 5.9–10.4) months, with 6-month and 10-month PFS rates of 67.3% and 29.6%, respectively. Median OS was 11.8 (95% CI 8.6–13.8) months, with 6-month, 10-month, and 12-month OS rates of 97.1%, 58.3%, and 48.8%, respectively (**Figure 2**). Median iPFS was 7.6 months for T-DM1 versus 10.9 months for T-DXd (P < 0.001); median PFS was 6.5 months versus 9.7 months (P = 0.021); median OS was 10.4 months versus 12.9 months (P = 0.046). For concurrent versus sequential treatment, median iPFS was 8.2 months versus 9.6 months (P = 0.091), median PFS was 8.0 months versus 7.4 months (P = 0.135), and median OS was 11.4 months versus 11.9 months (P = 0.724) (**Table 2**). Notable baseline imbalance across key prognostic covariates (standardized mean differences > 0.1) between T-DXd and T-DM1. We attempted multivariate Cox regression but the model failed to converge due to sparse events and imbalanced subgroups, thus adjusted HRs were not reported.

**Table 2:** Survival analysis of different subgroups.

Subgroups	iPFS (months)			PFS (months)			OS (months)		
	Median (95%CI)	Unadjusted HR (95%CI)	P value	Median (95%CI)	Unadjusted HR (95%CI)	P value	Median (95%CI)	Unadjusted HR (95%CI)	P value
ADCs category			<0.001			0.021			0.046
T-DM1	7.6 (5.5–8.3)	Reference		6.5 (4.5–9.0)	Reference		10.4 (9.0–11.2)	Reference	
T-DXd	10.9 (7.8–13.3)	0.37 (0.16–0.71)		9.7 (7.5–10.3)	0.46 (0.21–0.89)		12.9 (9.5–13.4)	0.52 (0.25–0.94)	
Treatment timing			0.091			0.135			0.724
Concurrent	8.2 (5.5–9.3)	Reference		8.0 (6.5–9.9)	Reference		11.4 (10.5–13.8)	Reference	
Sequential	9.6 (7.1–10.3)	0.70 (0.33–1.34)		7.4 (4.8–9.4)	1.24 (0.60–2.39)		11.9 (10.8–12.3)	1.08 (0.51–2.17)	

iPFS, intracranial progression−free survival; PFS, progression−free survival; OS, overall survival; HR, hazard ratios (unadjusted); CI, confidence interval; ADC, antibody−drug conjugate; T−DM1, trastuzumab emtansine; T−DXd, trastuzumab deruxtecan.

### 3.3 Short-term efficacy

At first assessment after ADC initiation, intracranial ORR was 74.3% (26/35) and intracranial DCR was 94.3% (33/35), including 10 cases of CR and 16 cases of PR. For T-DM1, intracranial ORR was 60.0% (6/10) and DCR 80.0% (8/10); for T-DXd, ORR was 80.0% (20/25) and DCR 100.0% (25/25). For concurrent treatment, intracranial ORR was 72.3% (16/22) and DCR 95.5% (21/22); for sequential treatment, ORR was 76.9% (10/13) and DCR 92.3% (12/13).

### 3.4 Safety

The most common treatment-related adverse events were decreased appetite, fatigue, leukopenia, anemia, nausea, vomiting, thrombocytopenia, and elevated transaminases, mostly grade 1–2. Grade ≥3 events were decreased appetite (23%), fatigue (11%), leukopenia (11%), and thrombocytopenia (9%). Among adverse events of special interest, radiation necrosis occurred in 9% (3/35), all were grade 1–2 and restricted to the concurrent T-DM1 subgroup; interstitial lung disease occurred in 3% (1/35), grade 1, in the T-DXd group (**Table 3**). This safety signal carries critical clinical implications: concurrent radiotherapy with T-DM1 increases the risk of grade 1–2 radiation necrosis, requiring regular brain enhanced MRI surveillance during combined treatment.

**Table 3:** Treatment-related adverse events.

Adverse event, n (%)	All patients (n=35)		T-DM1 (n=10)		T-DXd (n=25)	
	All Grades	Grade ≥3	All Grades	Grade ≥3	All Grades	Grade ≥3
Decreased appetite	30 (86)	8 (23)	9	3	21 (84)	5 (20)
Fatigue	28 (80)	4 (11)	8	5	20 (80)	3 (12)
Leukopenia	28 (80)	4 (11)	3	2	25 (100)	2 (8)
Anemia	24 (69)	2 (6)	7	0	17 (68)	3 (12)
Nausea	22 (63)	2 (6)	6	1	16 (64)	1 (4)
Vomiting	20 (57)	2 (6)	5	0	15 (60)	2 (8)
Thrombocytopenia	20 (57)	3 (9)	10	3	10 (40)	0 (0)
Elevated transaminases	12 (34)	2 (6)	3	0	9 (36)	2 (8)
Radiation necrosis	3 (9)	0 (0)	3	0	0 (0)	0 (0)
Interstitial lung disease	1 (3)	0 (0)	0	0	1 (4)	0 (0)

n, number; T-DM1, trastuzumab emtansine; T-DXd, trastuzumab deruxtecan.

## 4 Discussion

International guidelines recommend T-DXd as the preferred second-line regimen for HER2-positive advanced breast cancer, with T-DM1 also listed as a later-line option (12). In Chinese clinical practice and guidelines, pyrotinib plus capecitabine is the standard regimen for HER2-positive advanced breast cancer after trastuzumab failure, while T-DXd and T-DM1 are often recommended as subsequent options after TKI failure (11). T-DM1 continues to be a commonly used clinical option for Chinese breast cancer patients with brain metastases, largely driven by factors such as insurance coverage limitations, financial constraints, drug availability, and its earlier regulatory approval in China compared with T-DXd. This real-world study investigated the efficacy and safety of brain radiotherapy plus ADCs (T-DXd and T-DM1) in pretreated HER2-positive breast cancer patients with brain metastases.

Chinese clinical trials including PHENIX, PHOEBE, and PICTURE have demonstrated favorable PFS and high ORR with pyrotinib in advanced breast cancer (13–15). In patients with brain metastases, the PERMEATE trial showed promising efficacy of pyrotinib plus capecitabine (10). Due to these significant benefits, 86% of patients in this study received pyrotinib-based second-line anti-HER2 therapy before enrollment. Once new brain metastases or intracranial progression develops, brain radiotherapy and ADCs become important local and systemic treatments. WBRT and SRT are key local treatments, and SRT in particular yields favorable efficacy and low toxicity in patients with a limited number of brain metastases (16, 17). ADCs have also demonstrated robust intracranial activity. In the EMILIA and KAMILLA studies, T-DM1 improved outcomes in brain metastasis subgroups, with median PFS of 5.5 months and median OS of 18.9 months (18). Pooled analyses of DESTINY-Breast01, DESTINY-Breast02, and DESTINY-Breast03 showed that T-DXd resulted in median CNS PFS of 12.3 months in pretreated/stable brain metastases and 18.5 months in untreated/active brain metastases, and the DESTINY-Breast12 study, which enrolled 263 patients with brain metastases, reported median PFS of 17.3 months, intracranial ORR of 63.9%, and CNS PFS of 15.3 months (19). However, these trials included relatively few patients with prior TKI exposure, inconsistent with real-world Chinese clinical practice. A Chinese retrospective study showed favorable outcomes with T-DXd in patients with brain metastases after pyrotinib failure, with median CNS-PFS of 7.4 months and median OS of 9.8 months, but without combined brain radiotherapy (20). Studies investigating the efficacy and safety of brain radiotherapy plus ADCs remain limited, especially in TKI-pretreated populations consistent with Chinese clinical practice. A retrospective study included 16 patients with HER2-positive breast cancer brain metastases treated with SRT plus T-DM1 (completed within 6 months), reporting 1-year local control, PFS, and OS rates of 75%, 30%, and 67%, respectively (21). The largest global study of T-DXd plus radiotherapy enrolled 54 patients with metastatic breast cancer (HER2-positive and HER2-low), 25.9% receiving intracranial SRT, with excellent overall efficacy: 22.2% CR, 77.8% PR/SD, and near 100% DCR (22). An American retrospective study of 34 patients with breast cancer brain metastases treated with SRT plus T-DXd (within 3 months) reported 12-month local control of 97%, 24-month of 88%, median systemic PFS of 7 months, 12-month PFS rate of 32%, 24-month of 7%, 12-month OS rate of 64%, and 24-month of 53% (23). A similar Korean retrospective study of 29 patients with HER2-positive breast cancer brain metastases treated with SRT plus T-DXd reported 1-year any intracranial progression rate of 40%, local failure rate of 6.6%, and distant intracranial metastasis rate of 40% (24). These studies confirm the notable intracranial and systemic efficacy of brain radiotherapy plus ADCs, especially with T-DXd. Our study enrolled 35 patients with HER2-positive breast cancer brain metastases treated with brain radiotherapy (89% SRT) plus ADCs (71% T-DXd), with median iPFS of 8.4 months, median PFS of 7.9 months, median OS of 11.8 months, and intracranial ORR of 74.3%. These results provide preliminary support for the feasibility and potential efficacy of brain radiotherapy plus ADCs in TKI-pretreated patients.

In the head-to-head DESTINY-Breast03 trial, T-DXd significantly prolonged PFS (28.8 months vs 6.8 months) and improved ORR (78.5% vs 35.0%) compared with T-DM1 in patients with HER2-positive advanced breast cancer after trastuzumab failure, with consistent benefits in the brain metastasis subgroup (15.0 months vs 3.0 months) (25). Based on these compelling data, T-DXd has replaced T-DM1 as the standard second-line treatment for advanced breast cancer. In our subgroup analysis, T-DXd was associated with numerically longer survival endpoints in this small unadjusted cohort, and univariate log-rank comparisons demonstrated statistical differences without multivariable correction. We emphasize that these univariate survival differences should be interpreted as preliminary, unadjusted observational signals rather than as definitive evidence of therapeutic superiority. In addition, the univariate log-rank test detected no statistically significant survival differences between concurrent and sequential ADC-radiotherapy schedules, consistent with previous reports (23, 26).

The most common grade ≥3 adverse events in our study were decreased appetite (23%), fatigue (11%), leukopenia (11%), and thrombocytopenia (9%). Among adverse events of special interest, radiation necrosis occurred in 3 patients (9%) in the T-DM1 group, and interstitial lung disease occurred in 1 patient (3%) in the T-DXd group, all grade 1–2. Similar safety profiles have been reported in previous studies (22–24). Radiation necrosis was predominantly observed in the T-DM1 concurrent group (27–30). But the occurrence of three cases of radiation necrosis in this study limited the statistical power to identify independent risk factors for this complication, and this isolated interstitial lung disease cannot support any definitive conclusions regarding interstitial lung disease risk differences between T-DXd and T-DM1. The relatively low rate of radiation necrosis in our study may be related to the use of conventional WBRT, more fractionated SRT, and smaller lesions. One case of interstitial lung disease was noted in the T-DXd group, with overall manageable toxicity.

This study has several notable limitations, and its findings should be interpreted as preliminary rather than as definitive clinical evidence. As a single-center retrospective real-world analysis, it is inherently susceptible to selection, information and time biases. Treatment regimens (including ADCs type, radiotherapy timing, and radiotherapy regimens) were selected non-randomly based on clinical judgment and the background of the treatment era, which may confound subgroup comparisons of efficacy. Furthermore, the pooling of WBRT and SRT cohorts, although justified by their shared role as local therapy and by the study’s focus on ADC combination efficacy, may obscure modality-specific effects. The small sample size of 35 patients limits statistical power and precludes multivariate Cox regression analysis to identify independent prognostic factors, leaving potential unmeasured confounders unaccounted for. we acknowledge the absence of independent central blinded imaging review, which may have introduced subjective assessment bias in the classification of tumor responses. In addition, standardized prospective assessments of neurocognitive function and quality of life were not performed. For these reasons, our encouraging efficacy signals cannot be used to guide routine clinical practice at this time, and large-scale prospective multicenter randomized trials are urgently needed to confirm the intracranial benefits and long-term safety of this combination strategy. Despite these limitations, to our knowledge, this is the first real-world study to evaluate salvage brain radiotherapy combined with ADCs in heavily pyrotinib-pretreated Chinese patients with HER2-positive breast cancer and brain metastases, thereby providing unique population-specific data that expand the current literature on this understudied cohort.

## 5 Conclusion

In Chinese clinical practice, brain radiotherapy combined with either T-DXd or T-DM1 showed promising preliminary efficacy and tolerable safety signals in heavily pyrotinib-pretreated HER2-positive breast cancer patients with brain metastases in this retrospective single-center cohort. In this unadjusted retrospective cohort, patients receiving radiotherapy combined with T-DXd exhibited longer univariate survival outcomes compared with those treated with T-DM1. However, these univariate observational results are preliminary and require prospective multicenter validation with multivariate adjustment.

## Data Availability

The original contributions presented in the study are included in the article/supplementary material. Further inquiries can be directed to the corresponding author.
